# Unmet need for contraception among women in Benin: a cross-sectional analysis of the Demographic and Health Survey

**DOI:** 10.1093/inthealth/ihad049

**Published:** 2023-07-13

**Authors:** Paa Akonor Yeboah, Leticia Akua Adzigbli, Priscilla Atsu, Samuel Kwabena Ansong-Aggrey, Collins Adu, Abdul Cadri, Richard Gyan Aboagye

**Affiliations:** Fred N. Binka School of Public Health, University of Health and Allied Sciences, Hohoe, Ghana; Fred N. Binka School of Public Health, University of Health and Allied Sciences, Hohoe, Ghana; Department of Health Promotion, Education and Disability Studies, School of Public Health, Kwame Nkrumah University of Science and Technology, Kumasi, Ghana; Department of Health Policy, Management and Economics, School of Public Health, Kwame Nkrumah University of Science and Technology, Kumasi, Ghana; Center for Social Research in Health, UNSW Sydney, Sydney, NSW 2052, Australia; Department of Family Medicine, Faculty of Medicine, McGill University, Montreal, Quebec, Canada; Department of Social and Behavioural Science, School of Public Health, University of Ghana, Legon- Accra, Ghana; Fred N. Binka School of Public Health, University of Health and Allied Sciences, Hohoe, Ghana

**Keywords:** Benin, contraception, Demographic and Health Survey, unmet needs

## Abstract

**Background:**

The aim of the current study was to examine the prevalence and predictors of unmet need for contraception among women in sexual unions in Benin.

**Methods:**

Data for the study was extracted from the recent 2017–2018 Benin Demographic and Health Survey. A weighted sample of 9513 women of reproductive age was included in the study. We used multivariable multilevel binary logistic regression analysis to examine the factors associated with unmet need for contraception.

**Results:**

The prevalence of unmet need for contraception was 38.0% (36.7, 39.2). The odds of unmet need for contraception was higher among women with ≥4 births compared with those with no births, and among those who reported that someone else or others usually made decisions regarding their healthcare compared with those who make their own healthcare decisions. Wealth index was associated with a higher likelihood of unmet need for contraception. Also, the region of residence was associated with unmet need for contraception, with the highest odds being among women from the Mono region (adjusted odds ratio [aOR]=2.18, 95% CI 1.33 to 3.58).

**Conclusions:**

Our study shows that the unmet need for contraception among women in Benin is relatively high. Our findings call on relevant stakeholders, including government and non-governmental organisations, to enhance women's empowerment as part of interventions that seek to prioritise contraceptive services for women.

## Introduction

The use of contraceptives as a fertility regulator is widely accepted, and has an important role in women's health and well-being.^1^ Sustainable Development Goal (SDG) 3 which emphasizes achieving universal access to sexual and reproductive health services and lowering maternal mortality by 2030 highlights maternal health as a pressing issue.^2–4^ Women who are sexually active and fertile, but do not use any form of contraception and state that they do not want to have any more children or that they want to put off having children, are considered to have unmet need for contraception, according to the World Health Organization (WHO).^2^ These unmet demands contribute to unsafe abortions, increased fertility rates linked to poverty, high maternal death rates and low employment rates.^5^

In 2019, there were 1.9 billion women of reproductive age, out of which, an estimated 1.1 billion needed family planning services^6^; however, 270 million of these women had unmet contraception needs.^6^ Evidence from the recent projections indicated that by 2030, >10% of the world's unmet family planning needs will exist.^7^ Inadequate information on the use of contraceptives, their side effects, health issues, husbands' demands for certain behaviours and cultural or religious restrictions are all cited as the primary determinants of unmet need worldwide.^8,9^

Globally, sub-Saharan Africa has the highest proportion of women with unmet need for contraception, with approximately 25% (or about 47 million) of the region's women of reproductive age falling into this category.^10^ Overall, sub-Saharan Africa's sexual and reproductive health services have improved over time. However, further development is necessary to meet their demands in terms of reproductive health.^10^

Because of the desire for a large family size, common misunderstandings and misconceptions regarding the use of family planning services, the Republic of Benin (Benin) has historically had poor utilisation of family planning services, with a relatively low contraceptive prevalence of 14%.^11^ This has resulted in a high fertility rate in Benin.^11^ Family planning is encouraged by the government of Benin as part of its national health strategy. As of 2015, the goal of the country was to increase the usage of modern contraceptives among women of reproductive age from 9% in 2013 to 20% by 2018^12^; however, this could not be achieved. According to recent data, the utilisation of modern contraceptives is estimated to be 12%, while the total fertility rate for women between the ages of 15 and 49 y is 5.7%.^13^ According to Chae et al.^14^ 19% of all pregnancies in Benin were unplanned, and in 2017, both the newborn and maternal mortality rates were still high (397 per 100 000 live births and 30 per 1000 live births, respectively).^3,13^ To address this problem, a new goal has been set to increase modern contraceptive usage to 18% by 2026.^15^

To achieve this new goal, there is a need to critically examine factors that increase the likelihood of contraceptive usage among women in Benin. Considering the literary argument, prospects for addressing women's demands for contraception in Benin cannot be separated from their socioeconomic status, as well as important demographic traits; however, a gap in the literature exists regarding the individual and contextual level factors that predict the unmet need for contraception among women in Benin. This study, therefore, examined the prevalence and individual and contextual level factors associated with the unmet need for contraception among women in Benin.

## Materials and Methods

### Data source and study design

Data for the study was extracted from the 2017–2018 Benin Demographic and Health Survey (DHS). The DHS is a comparative, regularly conducted nationwide survey conducted across >90 countries to enhance the collection, analysis and dissemination of demographic, health and nutrition data, including family planning and unmet need for contraception.^16^ The DHS employs a cross-sectional design in collecting data from respondents.^17^ Respondents were sampled for the survey using a two-stage cluster sampling technique. A detailed sampling methodology has been highlighted in the literature.^17,18^ Structured questionnaires are used to collect data from the respondents using trained data collectors. Our study included 9513 married and cohabiting women with complete observations on all variables of interest. In drafting this paper, we relied on the Strengthening the Reporting of Observational Studies in Epidemiology (STROBE) guidelines.^19^ The dataset is freely accessible to download at https://dhsprogram.com/data/dataset/Benin_Standard-DHS_2017.cfm?flag=1.^20^

### Study variables

#### Outcome variable

Unmet need for contraception was the outcome variable in this study. In the DHS, unmet need for contraception consists of an aggregated sum of unmet need for spacing and limiting, and reproductive-age women who are married, fecund and/or sexually active have unmet need if they do not want any more children or want to postpone their next birth for at least 2 y but are not using contraception. Additionally, pregnant or amenorrhoeic women who had unwanted or mistimed pregnancies or births were considered unmet if they did not use contraception at the time they conceived. The women who had unmet need for contraception were coded as ‘1’, while those with no unmet need were coded as ‘0’. The categorisation employed in this study was informed by the literature.^21–25^

### Explanatory variables

We selected the explanatory variables for inclusion in the study based on their significant association with unmet need for contraception from the literature,^21–25^ as well as their availability in the DHS dataset. The variables were segregated into individual level and contextual level. The individual level variables consisted of a woman's age, level of education, marital status, religion, current working status, ethnicity, parity, frequency of watching television, frequency of listening to the radio, frequency of reading a newspaper or magazine, heard of family planning from a newspaper or magazine in the last few months, heard of family planning from the radio in the last few months, heard of family planning from television in the last few months and the person who usually decides on the respondent's healthcare. The contextual level variables included wealth index, gender of the head of the household, place of residence and region. In the DHS, the gender of the head of the household is coded as either male or female. Place of residence was ascribed to the location of the residence of the respondent and was considered either rural or urban based on the population metrics as stipulated in the DHS report.^16^ Wealth index was computed through principal component analysis of family and household assets such as the supply of drinking water, type of toilet facility, type of cooking fuel and possession of a television and fridge. Based on the individual rankings, households were categorised into five classes on the wealth index: poorest, poorer, middle, richer and richest. Detailed descriptions and coding of the wealth index have been highlighted in the literature.^16^

### Statistical analyses

We used Stata version 17.0 (Stata Corporation, College Station, TX, USA) to perform the analyses. Percentages were used to present the results on the sociodemographic characteristics and the prevalence of unmet need for contraception among the women. We adopted cross-tabulation to determine the distribution of unmet need for contraception across the explanatory variables. Later, binary logistic regression analysis was conducted to examine the explanatory variables statistically associated with unmet need for contraception. All the statistically significant variables were placed in the multilevel regression model. Subsequently, we employed a multivariable multilevel binary logistic regression analysis to examine the predictors of unmet need for contraception among the women in Benin using four models (Model O–III). Model O was an empty model with no explanatory variables. The result was used to show the variation in unmet need for contraception attributable to the primary sampling units. Model I contained the individual level variables, while Model II contained the contextual level variables. The final model (Model III) was considered the complete model and was built to contain the individual and contextual level variables. We used Akaike's information criterion (AIC) to check model fitness and comparability. Model III had the lowest AIC values; hence, it was selected as the best-fitting model. The multilevel regression results were presented as adjusted odds ratios (aORs), with their respective 95% confidence intervals (CIs). All the analyses were weighted to obtain unbiased estimates based on DHS guidelines.^16^

## Results

### Background characteristics of the respondents

Table 1 presents the results of the background characteristics of the women included in the study. The majority of the women were aged 25–29 y (26.7%), had no education (64.5%), were married (78.2%), were working (81.0%) and 50.2% were Christians. Most of the women belonged to the Fon ethnic group (33.9%), 45.7% had ≥4 births, 34.4% decided on their healthcare by themselves or with their partners, while 20.0% were of middle wealth index status. For mass media exposure variables, the majority (64.1%) of the women did not watch television, 43.2% did not listen to radio at all, and 93.7% did not read newspaper or magazine at all. Also, the majority of the women had not heard of family planning from newspapers or magazines (93.0%), radio (55.8%) and television (82.3%). Additionally, most women had a male as the head of their household (85.6%). Most of the women resided in rural areas (60.5%) and the majority (14.3%) were living in the Alibori region.

**Table 1.** Background characteristics of the respondents

Variable	Weighted N	Weighted %
Women's age (y)		
15–19	604	6.4
20–24	1929	20.3
25–29	2542	26.7
30–34	1878	19.7
35–39	1420	14.9
40–44	764	8.0
45–49	376	4.0
Level of education		
No education	6139	64.5
Primary	1745	18.4
Secondary or higher	1629	17.1
Marital status		
Married	7440	78.2
Cohabiting	2073	21.8
Religion		
Christianity	4784	50.2
Islamic	3175	33.4
African Traditional	928	9.8
None/others	625	6.6
Current working status		
No	1805	19.0
Yes	7708	81.0
Ethnicity		
Adja	1244	13.1
Bariba	1183	12.4
Dendi	585	6.2
Fon	3227	33.9
Yoa, Lokpa	286	3.0
Betamaribe	572	6.0
Peulh	934	9.8
Yoruba	980	10.3
Other Beninois	361	3.8
Other nationalities	141	1.5
Parity		
0 births	526	5.5
1 birth	1512	15.9
2 births	1598	16.8
3 births	1533	16.1
≥4 births	4344	45.7
Frequency of watching television		
Not at all	6104	64.1
Less than once a week	1567	16.5
At least once a week	1842	19.4
Frequency of listening to the radio		
Not at all	4113	43.2
Less than once a week	1998	21.0
At least once a week	3402	35.8
Frequency of reading a newspaper or magazine		
Not at all	8916	93.7
Less than once a week	356	3.8
At least once a week	241	2.5
Heard of family planning from a newspaper or magazine in the last few months		
No	8846	93.0
Yes	667	7.0
Heard of family planning from the radio in the last few months		
No	5305	55.8
Yes	4208	44.2
Heard of family planning from the television in the last few months		
No	7827	82.3
Yes	1686	17.7
Person who usually decides on the respondent's healthcare		
Respondent alone	917	9.6
Respondent and partner	3273	34.4
Partner alone	5261	55.3
Someone else or others	61	0.7
Wealth index		
Poorest	1863	19.6
Poorer	1882	19.8
Middle	1906	20.0
Richer	1979	20.8
Richest	1883	19.8
Gender of the head of the household		
Male	8146	85.6
Female	1367	14.4
Place of residence		
Urban	3756	39.5
Rural	5757	60.5
Region		
Alibori	1356	14.3
Atacora	775	8.1
Atlantic	1115	11.7
Borgou	1197	12.6
Collines	637	6.7
Couffo	638	6.7
Donga	622	6.5
Littoral	461	4.8
Mono	424	4.5
Ouémé	815	8.6
Plateau	573	6.0
Zou	900	9.5

### Bivariable analysis of unmet need for contraception among the respondents

The proportion of women with an unmet need for contraception was 38.0% (36.7, 39.2), as shown in Table 2. All the variables, except for the level of education, marital status, current working status, heard of family planning from newspapers or magazines in the last few months and heard of family planning on the television in the last few months, were statistically associated with unmet need for contraception from the crude regression analysis (Table 2).

**Table 2.** Prevalence of unmet need for contraception across the explanatory variables

Variable	Unmet need for contraception		
Prevalence	No (%) 62.0 (60.8, 63.3)	Yes (%) 38.0 (36.7, 39.2)	COR (95% CI)
Women's age (y)			
15–19	66.2 (61.9, 70.2)	33.8 (29.8, 38.1)	1.00
20–24	62.3 (59.8, 64.7]	37.7 (35.3, 40.2)	1.18 (0.97, 1.45)
25–29	64.8 (62.7, 66.8)	35.2 (33.2, 37.3)	1.06 (0.87, 1.30)
30–34	64.7 (62.1, 67.1)	35.3 (32.9, 37.9)	1.07 (0.87, 1.32)
35–39	59.9 (57.1, 62.6)	40.1 (37.4, 42.9)	1.31* (1.06, 1.62)
40–44	53.2 (49.0, 57.3)	46.8 (42.7, 51.0)	1.72*** (1.34, 2.21)
45–49	48.4 (42.9, 54.0)	51.6 (46.0, 57.1)	2.08*** (1.51, 2.86)
Level of education			
No education	62.4 (60.9, 63.9)	37.6 (36.1, 39.1)	1.00
Primary	59.7 (57.0, 62.3)	40.3 (37.7, 43.0)	1.12 (0.99, 1.26)
Secondary or higher	63.2 (60.5, 65.9)	36.8 (34.1, 39.5)	0.96 (0.84, 1.10)
Religion			
Christianity	59.1 (57.4, 60.8)	40.9 (39.2, 42.6)	1.00
Islamic	67.4 (65.1, 69.5)	32.6 (30.5, 34.9)	0.70*** (0.62, 0.79)
African Traditional	58.2 (54.8, 61.5)	41.8 (38.5, 45.2)	1.04 (0.90, 1.20)
None/others	63.0 (59.0, 66.9)	37.0 (33.1, 41.0)	0.85 (0.71, 1.02)
Marital status			
Married	62.3 (60.9, 63.8)	37.7 (36.2, 39.1)	1.0
Cohabiting	61.0 (58.4, 63.5)	39.0 (36.5, 41.6)	1.06 (0.94, 1.20)
Current working status			
No	61.4 (58.9, 63.9)	38.6 (36.1, 41.1)	1.00
Yes	62.2 (60.8, 63.6)	37.8 (36.4, 39.2)	0.97 (0.86, 1.09)
Ethnicity			
Adja	56.4 (52.8, 60.0)	43.6 (40.0, 47.2)	1.00
Bariba	70.4 (67.1, 73.5)	29.6 (26.5, 32.9)	0.54*** (0.44, 0.67)
Dendi	67.5 (61.7, 72.7)	32.5 (27.3, 38.3)	0.62** (0.45, 0.84)
Fon	59.1 (57.1, 61.0)	40.9 (39.0, 42.9)	0.90 (0.76, 1.06)
Yoa, Lokpa	61.9 (52.9, 70.1)	38.1 (29.9, 47.1)	0.80 (0.54, 1.19)
Betamaribe	58.4 (54.5, 62.3)	41.6 (37.7, 45.5)	0.92 (0.74, 1.14)
Peulh	69.5 (65.0, 73.6)	30.5 (26.4, 35.0)	0.57*** (0.44, 0.73)
Yoruba	56.6 (52.9, 60.3)	43.4 (39.7, 47.1)	0.99 (0.80, 1.23)
Other Beninois	73.3 (66.3, 79.2)	26.7 (20.8, 33.7)	0.47*** (0.33, 0.68)
Other nationalities	61.2 (53.0, 68.7)	38.8 (31.3, 47.0)	0.82 (0.57, 1.18)
Parity			
0 births	87.2 (83.7, 90.0)	12.8 (10.0, 16.3)	1.00
1 birth	63.2 (60.3, 66.1)	36.8 (33.9, 39.7)	3.96*** (2.89, 5.42)
2 births	63.1 (60.1, 66.0)	36.9 (34.0, 39.9)	3.98*** (2.90, 5.46)
3 births	62.8 (59.9, 65.6)	37.2 (34.4, 40.1)	4.03*** (2.96, 5.51)
≥4 births	57.9 (56.2, 59.7)	42.1 (40.3, 43.8)	4.94*** (3.69, 6.59)
Frequency of watching television			
Not at all	61.2 (59.7, 62.8)	38.8 (37.2, 40.3)	1.00
Less than once a week	62.4 (59.6, 65.2)	37.6 (34.8, 40.4)	0.95 (0.83, 1.08)
At least once a week	64.4 (61.9, 66.8)	35.6 (33.2, 38.1)	0.87* (0.77, 0.99)
Frequency of listening to the radio			
Not at all	60.3 (58.4, 62.1)	39.7 (37.9, 41.6)	1.00
Less than once a week	63.8 (60.8, 66.6)	36.2 (33.4, 39.2)	0.86* (0.75, 0.99)
At least once a week	63.2 (61.2, 65.1)	36.8 (34.9, 38.8)	0.88* (0.79, 0.99)
Frequency of reading a newspaper or magazine			
Not at all	61.7 (60.4, 62.9)	38.3 (37.1, 39.6)	1.00
Less than once a week	66.5 (60.9, 71.6)	33.5 (28.4, 39.1)	0.81 (0.64, 1.03)
At least once a week	69.0 (62.5, 74.8)	31.0 (25.2, 37.5)	0.72* (0.54, 0.97)
Heard of family planning from a newspaper or magazine in the last few months			
No	61.8 (60.5, 63.1)	38.2 (36.9, 39.5)	1.00
Yes	65.2 (61.3, 69.0)	34.8 (31.0, 38.7)	0.86 (0.72, 1.03)
Heard of family planning from the radio in the last few months			
No	60.3 (58.7, 61.8)	39.7 (38.2, 41.3)	1.00
Yes	64.3 (62.4, 66.1)	35.7 (33.9, 37.6)	0.84** (0.76, 0.93)
Heard of family planning from the television in the last few months			
No	61.5 (60.1, 62.9)	38.5 (37.1, 39.9)	1.00
Yes	64.4 (61.7, 67.1)	35.6 (32.9, 38.3)	0.88 (0.78, 1.00)
Person who usually decides on the respondent's healthcare			
Respondent alone	56.2 (52.6, 59.7)	43.8 (40.3, 47.4)	1.00
Respondent and partner	62.7 (60.8, 64.7)	37.3 (35.3, 39.2)	0.76** (0.65, 0.90)
Partner alone	62.8 (61.2, 64.4)	37.2 (35.6, 38.8)	0.76** (0.65, 0.89)
Someone else or others	45.1 (32.5, 58.5)	54.9 (41.5, 67.5)	1.56 (0.91, 2.68)
Wealth index			
Poorest	66.5 (64.0, 69.0)	33.5 (31.0, 36.0)	1.00
Poorer	61.2 (58.8, 63.5)	38.8 (36.5,41.2)	1.26** (1.09, 1.45)
Middle	60.5 (57.9, 63.0)	39.5 (37.0, 42.1)	1.30*** (1.12, 1.49)
Richer	61.0 (58.5, 63.4)	39.0 (36.6, 41.5)	1.27** (1.09, 1.48)
Richest	61.1 (58.5, 63.7)	38.9 (36.3, 41.5)	1.26** (1.08, 1.48)
Gender of the head of the household			
Male	62.7 (61.4, 64.0)	37.3 (36.0, 38.6)	1.00
Female	58.1 (55.0, 61.1)	41.9 (38.9, 45.0)	1.21** (1.06, 1.38)
Place of residence			
Urban	60.2 (58.2, 62.3)	39.8 (37.7, 41.8)	1.00
Rural	63.2 (61.6, 64.8)	36.8 (35.2, 38.4)	0.88* (0.79, 0.98)
Region			
Alibori	74.4 (71.0, 77.5)	25.6 (22.5, 29.0)	1.00
Atacora	62.0 (58.0, 65.9)	38.0 (34.1, 42.0)	1.78*** (1.40, 2.26)
Atlantic	55.9 (51.0, 60.6)	44.1 (39.4, 49.0)	2.29*** (1.77, 2.97)
Borgou	66.3 (62.6, 69.7)	33.7 (30.3, 37.4)	1.48** (1.17, 1.86)
Collines	58.2 (53.8, 62.5)	41.8 (37.5, 46.2)	2.08*** (1.63, 2.67)
Couffo	56.1 (51.9, 60.3)	43.9 (39.7, 48.1)	2.27*** (1.78, 2.89)
Donga	61.9 (56.1, 67.5)	38.1 (32.5, 43.9)	1.78*** (1.32, 2.40)
Littoral	58.2 (53.8, 62.4)	41.8 (37.6, 46.2)	2.08*** (1.63, 2.66)
Mono	56.3 (48.8, 63.4)	43.7 (36.6, 51.2)	2.25*** (1.60, 3.18)
Ouémé	59.3 (55.6, 62.9)	40.7 (37.1, 44.4)	1.99*** (1.59, 2.50)
Plateau	57.6 (52.5, 62.5)	42.4 (37.5, 47.5)	2.13*** (1.64, 2.78)
Zou	62.5 (59.4, 65.5)	37.5 (34.5, 40.6)	1.74*** (1.40, 2.15)

Abbreviation: COR, crude OR.

*p<0.05; **p<0.01; ***p<0.001; 1.00=reference category.

### Factors associated with an unmet need for contraception among the respondents

#### Fixed effect results

From Model III in Table 3, the age of the woman, frequency of listening to the radio, frequency of watching television, hearing of family planning on the radio in the last few months and those residing in rural areas, were associated with lower odds of an unmet need for contraception. The odds of unmet need for contraception increases as the number of births increases, with the highest odds among women with ≥4 births (aOR=8.77, 95% CI 6.21 to 12.40). Women who reported that it was someone else or others who usually make the decisions regarding their healthcare were more likely to report an unmet need for contraception (aOR=1.93, 95% CI 1.08 to 3.44) compared with those who made their own decisions. Wealth index was found to be associated with a higher likelihood of unmet need for contraception, with the highest odds among those in the richest quintile (aOR=1.38, 95% CI 1.11 to 1.72). Compared with women residing in Alibori, those in the other 11 regions had higher odds of an unmet need for contraception, with the highest odds among women from the Mono region (aOR=2.18, 95% CI 1.33 to 3.58).

**Table 3.** Multilevel analysis of factors associated with unmet need for contraception among women in Benin

Variable	Model O (empty model)	Model I AOR (95% CI)	Model II AOR (95% CI)	Model III AOR (95% CI)
Fixed effect results				
Women's age (y)				
15–19		1.00		1.00
20–24		0.74* (0.59, 0.94)		0.73** (0.58, 0.93)
25–29		0.48*** (0.37, 0.63)		0.46*** (0.36, 0.61)
30–34		0.41*** (0.31, 0.56)		0.40*** (0.30, 0.54)
35–39		0.48*** (0.35, 0.65)		0.46*** (0.33, 0.63)
40–44		0.64** (0.46, 0.90)		0.62** (0.44, 0.86)
45–49		0.75 (0.51, 1.11)		0.72 (0.48, 1.06)
Religion				
Christianity		1.00		1.00
Islamic		1.00 (0.84,1.19)		1.01 (0.85, 1.21)
African Traditional		0.93 (0.78, 1.11)		0.94 (0.79, 1.13)
None/others		0.90 (0.72, 1.11)		0.92 (0.74, 1.13)
Ethnicity				
Adja		1.00		1.00
Bariba		0.51*** (0.38, 0.69)		0.76 (0.51, 1.13)
Dendi		0.50*** (0.35, 0.72)		0.74 (0.47, 1.16)
Fon		0.88 (0.72, 1.07)		0.96 (0.73, 1.28)
Yoa, Lokpa		0.82 (0.55, 1.23)		1.07 (0.64, 1.77)
Betamaribe		0.76 (0.57, 1.02)		1.02 (0.64, 1.62)
Peulh		0.55*** (0.40, 0.76)		0.84 (0.55, 1.30)
Yoruba		1.01 (0.78, 1.30)		1.18 (0.83, 1.69)
Other Beninois		0.44*** (0.30, 0.65)		0.63 (0.39, 1.01)
Other nationalities		0.82 (0.53, 1.26)		0.92 (0.58, 1.47)
Parity				
0 births		1.00		1.00
1 birth		4.79*** (3.45, 6.67)		4.81*** (3.47, 6.69)
2 births		5.68*** (4.02, 8.02)		5.74*** (4.07, 8.09)
3 births		6.47*** (4.60, 9.10)		6.63*** (4.72, 9.32)
≥4 births		8.41*** (5.95, 11.87)		8.77*** (6.21, 12.40)
Frequency of watching television				
Not at all		1.00		1.00
Less than once a week		1.02 (0.88, 1.19)		0.99 (0.85, 1.15)
At least once a week		0.91 (0.78, 1.05)		0.83* (0.70, 0.98)
Frequency of listening to the radio				
Not at all		1.00		1.00
Less than once a week		0.79** (0.68, 0.93)		0.78** (0.67, 0.91)
At least once a week		0.88 (0.76, 1.02)		0.86* (0.74, 1.00)
Frequency of reading a newspaper or magazine				
Not at all		1.00		1.00
Less than once a week		0.91 (0.70, 1.19)		0.89 (0.69, 1.17)
At least once a week		0.83 (0.59, 1.15)		0.80 (0.57, 1.11)
Heard of family planning from the radio in the last few months				
No		1.00		1.00
Yes		0.84** (0.75, 0.95)		0.83** (0.74, 0.94)
Person who usually decides on the respondent's healthcare				
Respondent alone		1.00		1.00
Respondent and partner		0.84 (0.69, 1.01)		0.84 (0.70, 1.01)
Partner alone		0.92 (0.78, 1.10)		0.95 (0.80, 1.13)
Someone else or others		1.95* (1.09, 3.47)		1.93* (1.08, 3.44)
Wealth index				
Poorest			1.00	1.00
Poorer			1.17* (1.01, 1.35)	1.22* (1.05, 1.42)
Middle			1.19* (1.03, 1.37)	1.30** (1.11, 1.52)
Richer			1.05 (0.90, 1.23)	1.26** (1.06, 1.50)
Richest			0.96 (0.80, 1.16)	1.38** (1.11, 1.72)
Gender of the head of the household				
Male			1.00	1.00
Female			1.08 (0.94, 1.24)	1.08 (0.94, 1.24)
Place of residence				
Urban			1.00	1.00
Rural			0.89 (0.79, 1.02)	0.85* (0.75, 0.98)
Region				
Alibori			1.00	1.00
Atacora			1.99*** (1.54, 2.58)	1.70** (1.24, 2.35)
Atlantic			2.47*** (1.89, 3.23)	2.16*** (1.46, 3.19)
Borgou			1.53*** (1.20, 1.97)	1.42* (1.08, 1.86)
Collines			2.23*** (1.72, 2.90)	1.67** (1.17, 2.38)
Couffo			2.43*** (1.87, 3.15)	2.12** (1.35, 3.34)
Donga			1.83*** (1.38, 2.42)	1.58** (1.14, 2.17)
Littoral			2.33*** (1.76, 3.09)	2.09*** (1.45, 3.01)
Mono			2.49*** (1.73, 3.60)	2.18** (1.33, 3.58)
Ouémé			2.13*** (1.66, 2.74)	1.62** (1.13, 2.33)
Plateau			2.25*** (1.70, 2.97)	1.69** (1.15, 2.49)
Zou			1.89*** (1.50, 2.38)	1.67** (1.16, 2.40)
Random effect model				
Primary sampling unit variance (95% CI)	0.318 (0.255, 0.397)	0.279 (0.221, 0.353)	0.239 (0.189, 0.304)	0.249 (0.196, 0.315)
Intraclass correlation	0.088	0.078	0.068	0.070
Wald χ^2^	Reference	356.41 (<0.001)	98.10 (<0.001)	430.26 (<0.001)
Model fitness				
Log-likelihood	-10322.845	-9985.7847	-10272.037	-9949.4393
Akaike information criterion	20 649.69	20 039.57	20 582.07	20 000.88
Total sample used	9513	9513	9513	9513
Number of clusters	555	555	555	555

Abbreviation: AOR, adjusted OR.

*p<0.05; **p<0.01; ***p<0.001; 1.00=reference category.

#### Random effect results

Results from Table 3, Model O, showed that unmet need for contraception varies significantly across the clusters (σ2=0.318, 95% CI 0.255 to 0.397). The results in Model O revealed that the between-cluster variations accounted for approximately 9% of the prevalence of unmet need for contraception (intraclass correlation [ICC]=0.088). The between-cluster variation decreased to 8% in the model containing only individual-level variables (Model I). Further reduction in the ICC value was found in Model II and then increased slightly to 7% in Model III. This result shows that the variances across the clusters could account for the observed difference in the unmet need for contraception among the women in Benin.

## Discussion

This study examined the prevalence and factors associated with unmet need for contraception among women in Benin. In this study, we found that the prevalence of unmet need for contraception was 38.0%. The prevalences found in similar studies conducted in other African countries—35.17% in Ghana,^26^ 32.2% in Papua New Guinea^21^ and 32.4% in Burundi^27^—were slightly below that of the current study. The relatively higher proportion of unmet need for contraception in this study may be attributable to the differences in the cultural, social, economic and demographic characteristics of women across the various study settings.^28^

The current study revealed a positive association between unmet need for contraception and parity. Thus, women with one, two, three, and four or more children had a high unmet need for contraception. The results from this study agree with those reported from studies conducted in sub-Saharan Africa,^22^ low-and-middle-income countries,^29^ Papua New Guinea^21^ and Nigeria,^30^ which reported that the unmet contraceptive need of a woman increases as the number of births increases. Unlike the aforementioned studies, those conducted in Egypt,^31^ Ethiopia^32^ and Zambia^33^ showed an inverse relationship between the unmet need for contraception and parity. The positive relationship between parity and the unmet need for contraception is detrimental to maternal and child health. Therefore, communication about additional contraception and educational programmes for women who attend postnatal care are imperative.

We found that wealth status increases the likelihood of an unmet need for contraception, with those women belonging to the richest wealth index reporting 38% higher odds of unmet need for contraception compared with those in the poorest wealth index. This result agrees with those reported from earlier studies conducted in Ethiopia, which also found a positive relationship between wealthy women and unmet need for contraception.^32–34^ However, this result varies from previous studies conducted in Nigeria,^35^ Burundi,^27^ Papua New Guinea^21^ and sub-Saharan Africa,^22^ which stated that the wealth index and unmet need for contraception are inversely related. These observations require further inquiry to understand the contexts in which the wealth index may be positively or negatively associated with unmet need for contraception among women.

In this study, women who utilised mass media (i.e. watched television, listened to the radio) at least once a week were less likely to have an unmet need for contraception. Those who heard about family planning on the radio in the last few months were also protected against unmet need for contraception. These results are in line with a study conducted in Burundi, which stated that women exposed to radio or television were less likely to have an unmet need for contraception.^27^ This finding reiterates the importance of using mass media as a medium for behavioural change communication.^36^ Because women who are exposed to mass media, public education and advertisements for contraceptives will be knowledgeable, they will therefore make informed decisions using the deeper understanding they have gained regarding the need for contraception.^22^

Compared to women from Alibori region, those from the other regions in Benin had higher odds of unmet need for contraception. This finding draws attention to the requirement to increase efforts to achieve health equity in the context of access to contraceptives and their use in Benin. Access may be different across the regions, as evidenced by the differences observed in the current study. This result calls for further investigation to understand the reasons for the differences in odds across the regions. Another plausible reason for the observed finding could be the disparities in the population sizes of women in rural and urban areas, with women in the Alibori region mostly residing in rural areas. However, the high literacy rate could account for the increased unmet need for contraception among women from other regions (except for Alibori).

In this study, it was found that women who resided in rural areas were 15% less likely to have an unmet need for contraception. This finding is similar to those reported by studies conducted in Indonesia^37^ and Nigeria,^38^ which also revealed that women in rural areas had lower odds of an unmet need for contraception compared with women residing in urban areas. Contrary to this finding are results from other studies in other developing countries like Papua New Guinea^21^ and Burundi,^27^ where women who resided in a rural area had a higher likelihood of an unmet need for contraception. This difference could potentially highlight the gap in access to health services in urban and rural areas in most parts of sub-Saharan Africa (including Benin), thus supporting the call for increased efforts towards achieving universal health coverage and health equity.

Lastly, we found that healthcare decision-making was associated with unmet need for contraception, with women whose healthcare decisions were made by someone else or others reporting higher odds of unmet needs for contraception compared with women who made their own healthcare decisions. This finding is similar to those reported by studies in Senegal^39^ and Burkina Faso,^40^ which also revealed that healthcare decision-making was associated with unmet need for contraception. This finding reiterates the need to encourage autonomy in health decision-making among women in Benin. To achieve this, it is important to understand the sociocultural and economic factors in Benin, including gender roles and relations, and how they affect women's autonomy in healthcare decision-making.^41^

### Strengths and limitations of the study

A major strength of the current study is its use of a national dataset, which was used to examine the unmet need for contraception and associated factors among women in Benin. The larger sample size used in the study helps to increase the precision of the estimates made, hence increasing the likelihood of generalisability and the validity of the findings reported in the study. The results of the current study provide a stepping stone for further deliberation on specific strategies to adopt to increase the likelihood of attaining the current contraceptive goal in Benin by 2026. Conversely, there is an element of recall bias in the study, as women may not vividly remember previous experiences. Also, some women may prefer to give socially acceptable responses so as not to be associated with any type of stigma or perception. Additionally, the rural–urban population dynamics that constitutes each region could have influenced the study's findings.

### Public health and policy implications

The findings of this study call for actions from relevant stakeholders, including the Ministry of Health, non-governmental organisations and women's groups, among others, to improve the availability of contraception and its usage for women in Benin. The government of Benin, as part of its strategies to achieve universal health coverage and SDG 3, can use these results to guide conversations around strategies to adopt to intensify the provision of contraceptive service delivery and usage among women. As part of the strategies to reduce the unmet need for contraception in Benin, the results of this study call for relevant stakeholders to make good use of mass media to effect sustained behavioural change and utilisation of contraceptive services. The study’s results further call for intense actions to empower women and to take action to disentangle gender roles and relations that increase the odds of unmet need for contraception.

## Conclusion

The current study indicates that unmet need for contraception among women in Benin is relatively high. The study found that parity, region of residence, wealth index, mass media exposure, healthcare decision-making and area of residence were significantly associated with unmet need for contraception among women in Benin. Therefore, programmes and interventions must take into consideration the key findings of this study; most importantly, there is a need to strengthen efforts toward women's empowerment in Benin.

## Data Availability

The dataset is freely accessible at https://dhsprogram.com/data/dataset/Benin_Standard-DHS_2017.cfm?flag=1.
